# Optimization of an acid digestion procedure for the determination of Hg, As, Sb, Pb and Cd in fish muscle tissue

**DOI:** 10.1016/j.mex.2017.11.006

**Published:** 2017-11-11

**Authors:** Elisabeth Mohammed, Terry Mohammed, Azad Mohammed

**Affiliations:** a Department of Chemistry, University of the West Indies, St Augustine, Trinidad and Tobago; b Department of Life Sciences, University of the West Indies, St Augustine, Trinidad and Tobago

**Keywords:** Acid digestion of fish tissue, Heavy Metals, Digestion, Optimization

## Abstract

A simple, efficient and cost effective method was developed and optimized for the digestion of fish tissue for the determination of Lead, Cadmium, Mercury, Antimony, Arsenic and Selenium by FAAS, CVAAS and HGAAS. Three of the more common classical open tubed acid digestion procedures were explored with the purpose of optimizing the variables and selecting the single most efficient, convenient and inexpensive digestion method. The effect of parameters such as digestion media, digestion time and digestion temperature on the efficiency of extraction of heavy metals from fish tissue was examined. Concentrated nitric acid was determined to be the most efficient digestion media for all the metals studied.

• Efficient extraction of Arsenic, Selenium, Antimony, Lead and Cadmium was achieved at a digestion temperature of 100 °C for 120 min.
• Optimum extraction of Mercury in fish tissue was achieved at 85 °C for 120 min since at higher temperatures, mercury was lost due to volatilization.

## Graphical abstract

**Figure:**
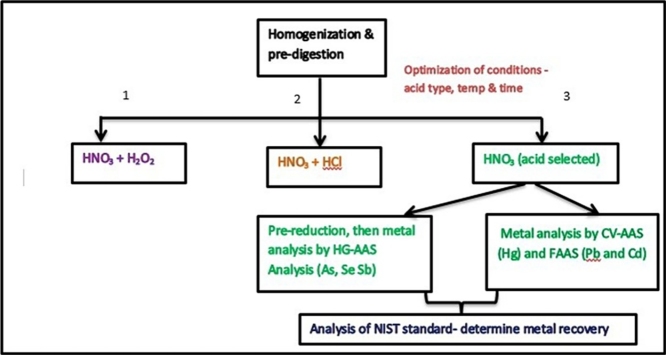

## Method details

This procedure describes a low cost open digestion method. The main advantages of this method are superior extraction efficiency, speed, ability to analyze multiple elements including volatile elements, ability to digest large volumes of samples and reduced cost. Many researchers use microwave digestion techniques to solubilize biological samples [1], [2], however, without such costly equipment available, open acid digestion presents the most viable option. There exists no single open acid digestion procedure for the analysis of metals for all biological materials [3]. The nature of the biological sample, the analyte, the reagent availability and equipment usually play a decisive role in the selection of the digestion procedure because it helps to select the best conditions suitable to give the highest yield of extractable metals.

The variables to be optimized include:

1. Digestion reagents
2. Digestion temperature
3. Digestion time

The selection of digestion reagents depends on the sample matrix and the metals being analyzed. Xiao [3], suggested that the most effective acids used in digestions of inorganic and organic samples are HCl, H_2_SO_4_, HF and HNO_3_ because of their strong oxidizing ability. Their oxidizing strength can be increased by the addition of chlorate, permanganate and hydrogen peroxide. Nitric acid is the most commonly used acid for oxidation of organic matrices. Its oxidizing strength can be increased when used in conjunction with other acids. Although hydrochloric acid is a non-oxidizing acid when used alone, when mixed with nitric acid (aqua regia) in a certain proportion (HCl:HNO_3_ 1:3 ratio), it becomes a strong oxidizing agent. This is because the products of their reaction, nitrosyl chloride and chlorine are strong oxidizing agents [3]. Some metals form insoluble precipitates with the anion of the acid therefore some acids are totally incompatible with certain metals [4].

The digestion efficiency can also be influenced by other factors such as temperature and digestion time. The digestion temperatures of open acid digestion systems are determined by the boiling points of the acids and the volatility of the analyte [5]. Temperature increases the average kinetic energy resulting in increased collisions between the acid and biological sample which enhances the dissolution of metals [6]. However, when heat is applied to the vessel containing the solution it usually takes time for the solution to begin to heat up. The digestion process is also limited by the boiling point of the acid or acids used. In multi-acid digestion, the most volatile acids boil away first followed by the next most volatile [7]. The boiling point of nitric acid, aqua regia and HNO_3_: H_2_O_2_ (1:1) are 120, 108 and 85 °C respectively. High temperatures, in excess of the boiling point of the acid or acid mixture, can result in a loss of volatile metals such as mercury and lead [6].

Digestion time is another important factor because it controls the length of exposure of the matrix to the oxidizing acid. The length of exposure can increase exothermic processes which will increase the extent of solubilization of the metal of interest from the biological matrix as well as the loss of these metals through volatilization [8]. The typical time to complete a wet digestion by open vessel digestion is from 1 to 2 h [9] but can be longer depending on specific conditions. Therefore, it is important to ensure that the most efficient digestion temperature and time is selected to obtain the best recovery of the element.

A simple, efficient and cost effective method had to be adopted because a large quantity of fish muscles samples needed to be analyzed hence the purpose of this study were to optimize the extraction of a range of metals by:

1. Obtaining the most efficient and cost-effective combination of digestion media, digestion time and digestion temperature that would completely extract all the metals of interest.
2. Select the method that minimizes the loss of the metals of interest.
3. Select the method with the least possibility of contamination or losses.

## Sampling/sample preparation and digestion

Materials

• Polyethylene bags, (Ziploc, USA)
• Ice
• Stainless steel food processor (Hamilton Beach)
• 65% Nitric acid, 37% Hydrochloric acid and 30% Hydrogen peroxide- ACS grade, (Sigma-Aldrich, USA)
• Whatman 541 filter paper, (Sigma-Aldrich, USA)
• Heating block (VWR reaction + heating block)- capable of exceeding 150 °C, (VWR, USA)
• Acid washed volumetric flasks (50 mL-Class A) and (25 mL-Class A), (Pyrex, USA)
• 50 mL Pyrex boiling tubes, (Pyrex, USA)

Shark samples were obtained from Maracas Bay on the North Coast of Trinidad and transported on ice to the laboratory where they were stored at −20 °C until required for analysis. 0.9 kg of shark sample was thawed at room temperature, the skin and bone tissue were removed then homogenized using a food processor. The homogenized tissue was subsampled and 0.5 g aliquots samples weighed (to the nearest 0.1 mg) into dried boiling tubes.

Acids were added in the following combinations:

• 10 mL of concentrated HNO_3_–90 tubes
• 10 mL of acid mixture concentrated HNO_3_/HCl (3:1) –90 tubes
• 10 mL of acid mixture concentrated HNO_3_/H_2_O (1:1) –90 tubes

Samples were predigested at room temperature for 24 h (26 °C) then digested on a heating block at different temperatures ranging from 60 to 150 °C (Table 1). Samples were then cooled and filtered through Whatman No. 541 filter paper. The tubes and filters were rinsed, and the filtrate made up to 50 mL with de-ionized water, and stored in volumetric flasks at 4 °C until analysis.

**Table 1:** Variables (acid, temperature and time) optimized for acid digestion.

Metal	Acid Combination	Temperature/°C	Time/min
Mercury	HNO_3_	25	30, 60, 90, 120 and 150
Arsenic		60	30, 60, 90, 120 and 150
Antimony		85	30, 60, 90, 120 and 150
Selenium		100	30, 60, 90, 120 and 150
Lead		130	30, 60, 90, 120 and 150
Cadmium		150	30, 60, 90, 120 and 150
	HNO_3_: HCl	25	30, 60, 90, 120 and 150
		60	30, 60, 90, 120 and 150
		85	30, 60, 90, 120 and 150
		100	30, 60, 90, 120 and 150
		130	30, 60, 90, 120 and 150
		150	30, 60, 90, 120 and 150
	HNO_3_: H_2_O_2_	25	30, 60, 90, 120 and 150
		60	30, 60, 90, 120 and 150
		85	30, 60, 90, 120 and 150
		100	30, 60, 90, 120 and 150
		130	30, 60, 90, 120 and 150
		150	30, 60, 90, 120 and 150

Additional treatment steps were required for arsenic, antimony, selenium and mercury following digestion. Mercury had to be reduced to the ground state atoms while arsenic, antimony and selenium had to be converted to the respective hydrides before analysis. For both arsenic and antimony, 5 mL aliquots of the digests were pipetted into boiling tubes and 10 mL of a 1% potassium iodide in 1 M HCl added to further reduce the digests. They were then heated for 4 min at 70 °C, cooled and filtered through Whatman No. 541 filter paper, after which they were made up to 25 mL with de-ionized water.

**Fig. 1:**
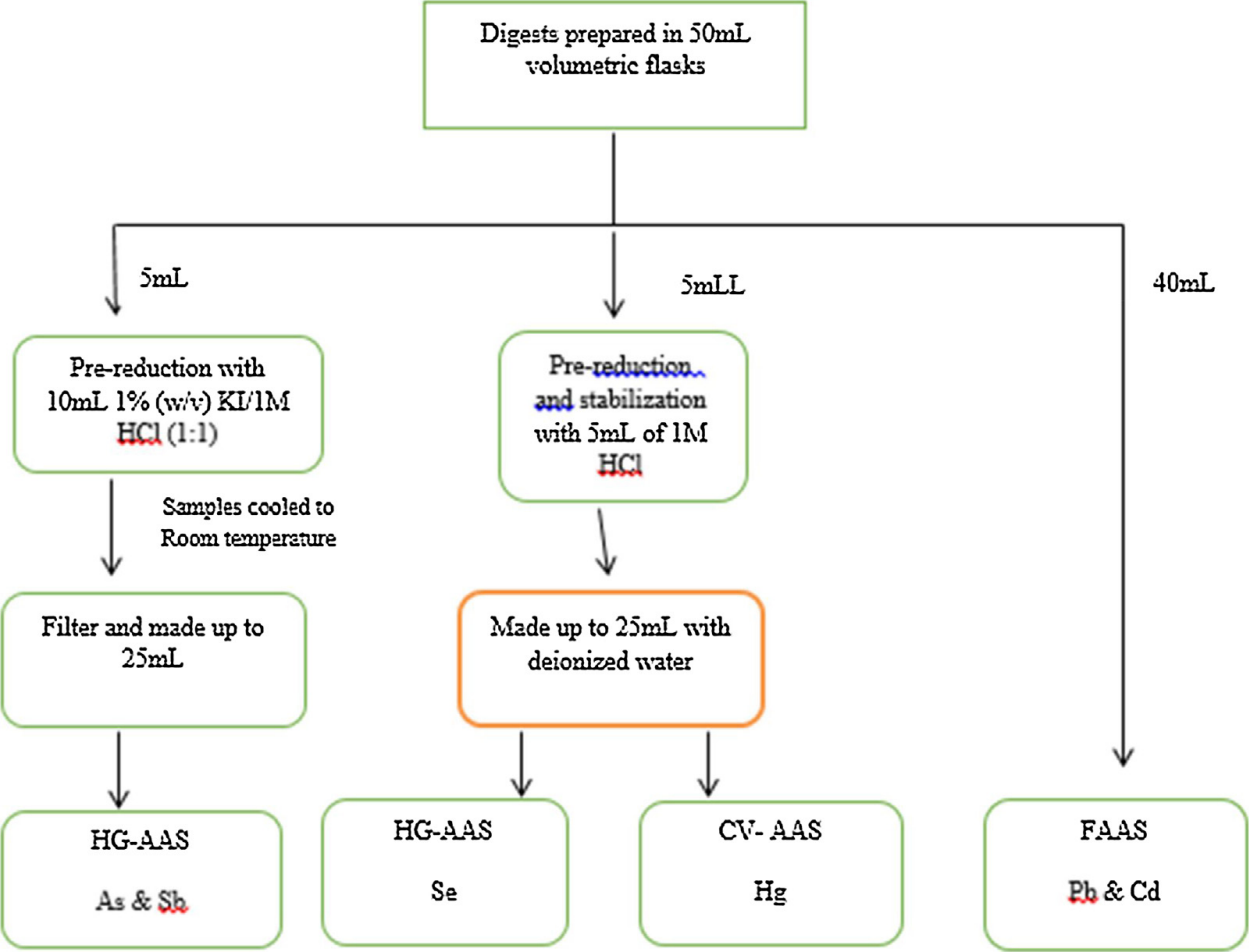
Flowchart outlining pre-reduction and stabilization steps between acid digestion and sample analysis.

Although mercury and selenium were analyzed by different techniques (CVAAS vs HGAAS) the samples were prepared in a similar manner. 5 mL aliquots of the digests were pipetted into boiling tubes and 5 mL of 1 M HCL were added to each, the resulting solutions were made up to 25 mL with deionized water. The acid blank digests and spiked samples were processed in a similar manner. See Fig. 1 for an outline of the above procedure.

## Flame AAS/CV-AAS/HG-AAS analysis

Instrumentation and Materials

• SpectrAA 880 Varian Atomic Absorption Spectrophotometer (Varian Inc. USA)
• Agilent vapor generation accessory VGA-77 (Agilent technologies Inc. USA)
• AccuStandard stock solutions of mercury (1000 μg/mL), arsenic (1000 μg/mL), lead (1000 μg/mL) and cadmium (1000 μg/mL) (Accustandard, USA)
• Inorganic Ventures stock solutions of selenium (1000 μg/mL) and antimony (1000 μg/mL) (Inorganic Ventures, USA).
• Tin (II) chloride, reagent grade, 98%, (Sigma-Aldrich, USA)
• Sodium borohydride, ACS grade, (Sigma-Aldrich, USA)
• Sodium hydroxide, ACS grade, (Sigma-Aldrich USA)
• Hydrochloric acid, ACS grade, (Sigma-Aldrich, USA)
• Potassium iodide, ACS grade, (Sigma-Aldrich, USA)
• Hot plate, VWR reaction + heating block, (VWR, USA)

The specific atomic absorption spectrophotometry technique and the corresponding conditions used to achieve the best analytical sensitivity and precision for each metal is shown in Table 2. The reducing agents, stannous chloride and sodium borohydride were prepared daily. Since arsenic, antimony, mercury and selenium were both being determined on the same sample, mercury and selenium were determined first to avoid cross-contamination from the potassium iodide used in the reduction process for Antimony and Arsenic. Potassium iodide has the potential to suppress the analytical signal for these metals [10]. Results obtained from the Atomic Absorption Spectrophotometer were in μg/L, and were converted to μg/g in order to evaluate the method efficiencies. Each sample was analyzed in triplicate and the results were reported as mean ± standard deviation (SD) on a wet weight basis.

**Table 2:** Instrumental operating conditions of Flame-AAS, CV-AAS and HG-AAS.

Analytical Technique	Flame-AAS	CV-AAS	HG-AAS	
Elements	Pb, Cd	Hg	As, Sb	Se
Flame type	Air/acetylene	NA	Air/Acetylene	Air/Acetylene
Reductant	NA	SnCl_2_ (25% w/v)/HCl (15% v/v)	NaBH_4_ (0.6% w/v)/NaOH (0.5% v/v)	NaBH_4_ (0.6% w/v)/NaOH (0.5% v/v)
Pre-reductant	NA	NA	1% KI/1 M HCl	1 M HCl
Acid	NA	De-ionized water	5 M HCl	5 M HCl

## Quality assurance–metal recovery

Materials

• Dorm-4 Certified Reference Fish Protein, NIST Traceable (National Research Council, Canada).
• Nitric acid, ACS grade, ((Sigma-Aldrich, USA)
• 1000 ppm Pb, Cd, Hg, As, (Accustandards, USA), Sb and Se, (Inorganic Ventures, USA) Atomic Absorption Spectrophotometry Standard

In order to evaluate the accuracy of the acid digestion method 0.5 g samples of the Dorm-4 NIST standard were digested in triplicate under the optimized conditions (acid, temperature and time) selected for each metal and analyzed by AAS, CV-AAS and HG-AAS. The results obtained were compared with certified results, and recoveries determined.

In addition to the use of a Certified Reference Material, spiked sample recoveries were also determined for each metal. 0.5 g aliquots of homogenized fish tissue were spiked with a working standard (100 ppm) derived from the 1000 ppm stock standard. Sufficient standard was added to the sample using a micro pipette such that the final volume after digestion (50 mL) contained an additional 5 ppm lead, 1 ppm cadmium, and 30 ppb of mercury, arsenic, antimony and selenium. The recoveries were determined.

## Discussion

**Fig. 2:**
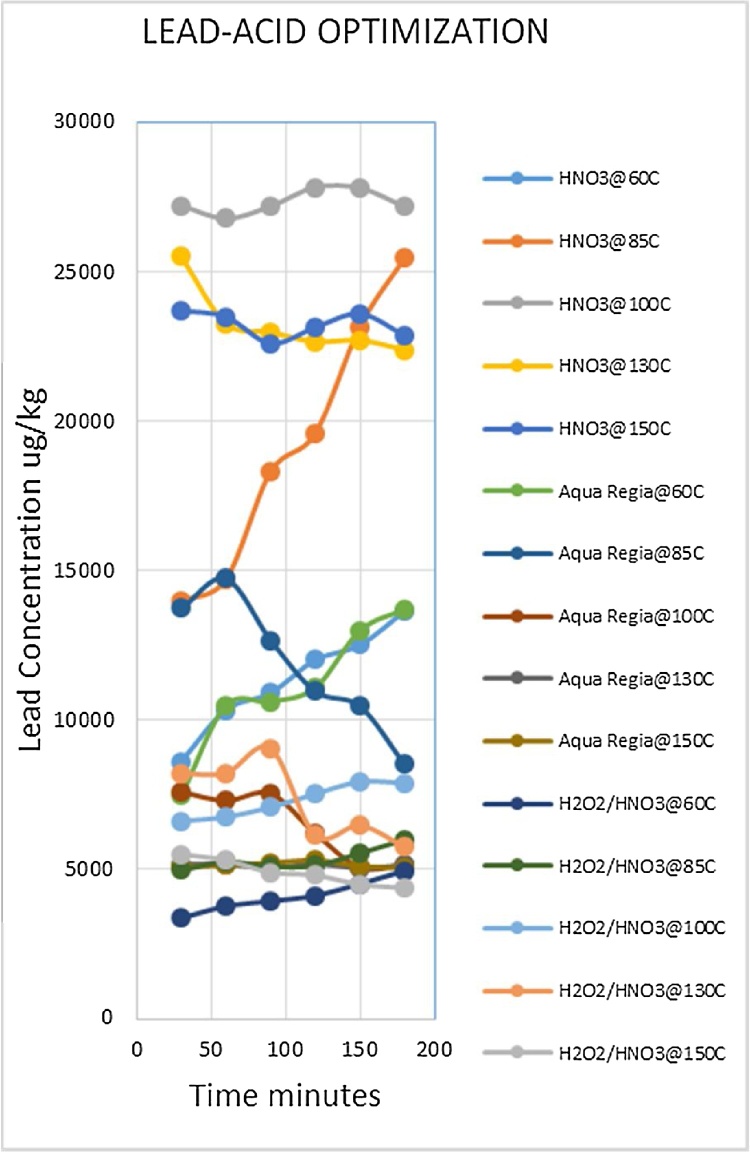
Optimization of Acid digestion for Lead.

**Fig. 3:**
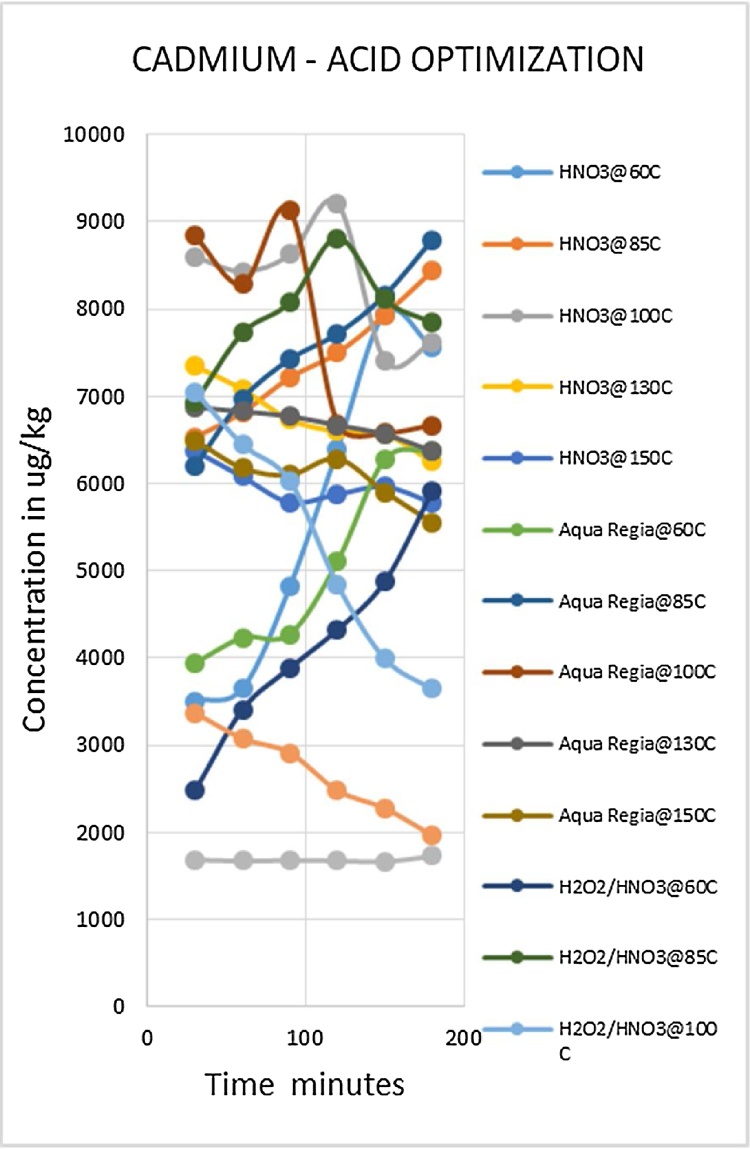
Optimization of Acid digestion for Cadmium.

**Fig. 4:**
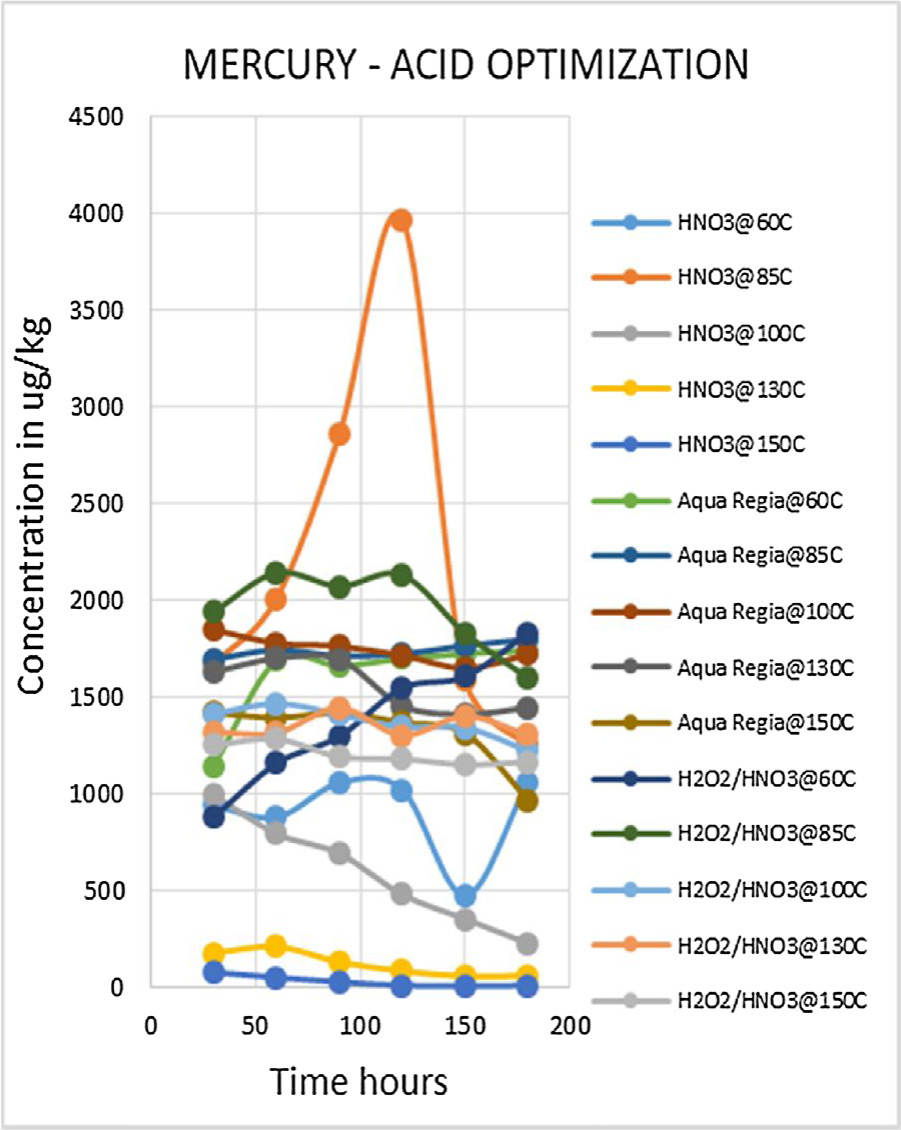
Optimization of Acid digestion for Mercury.

**Fig. 5:**
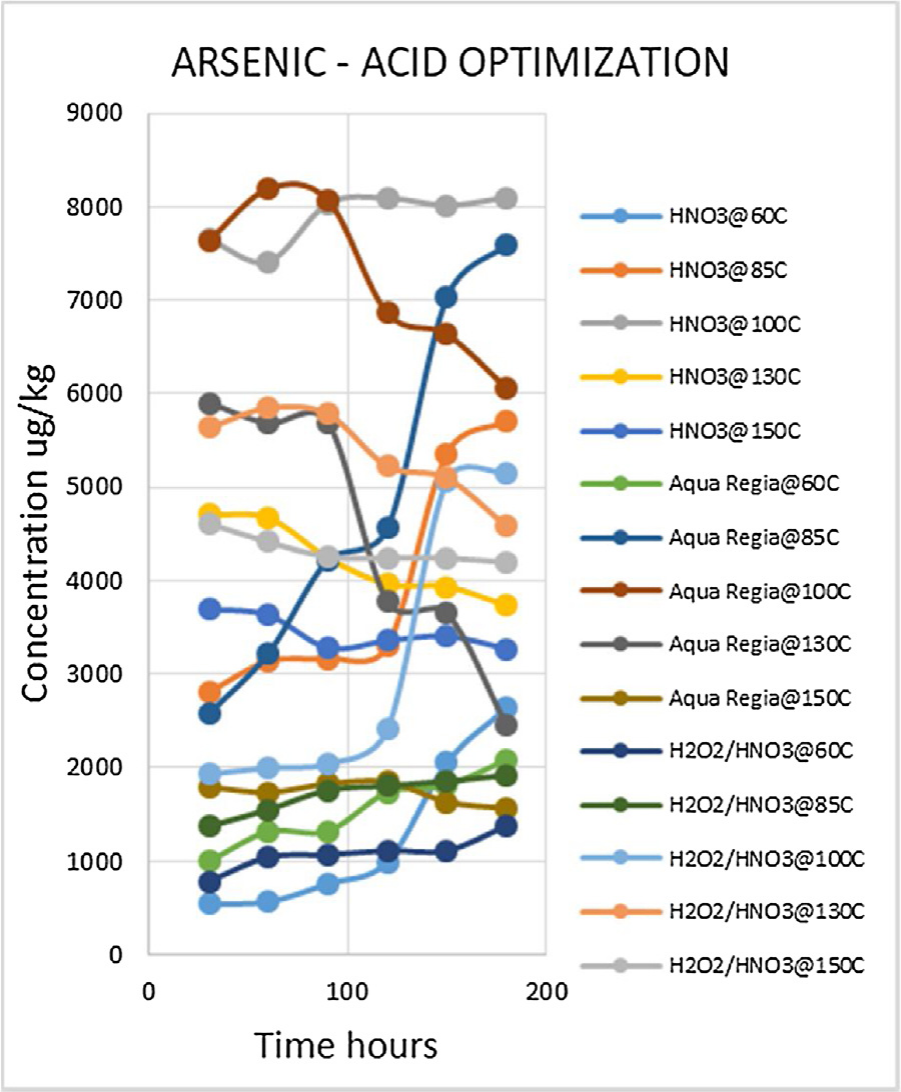
Optimization of Acid digestion for Arsenic.

**Fig. 6:**
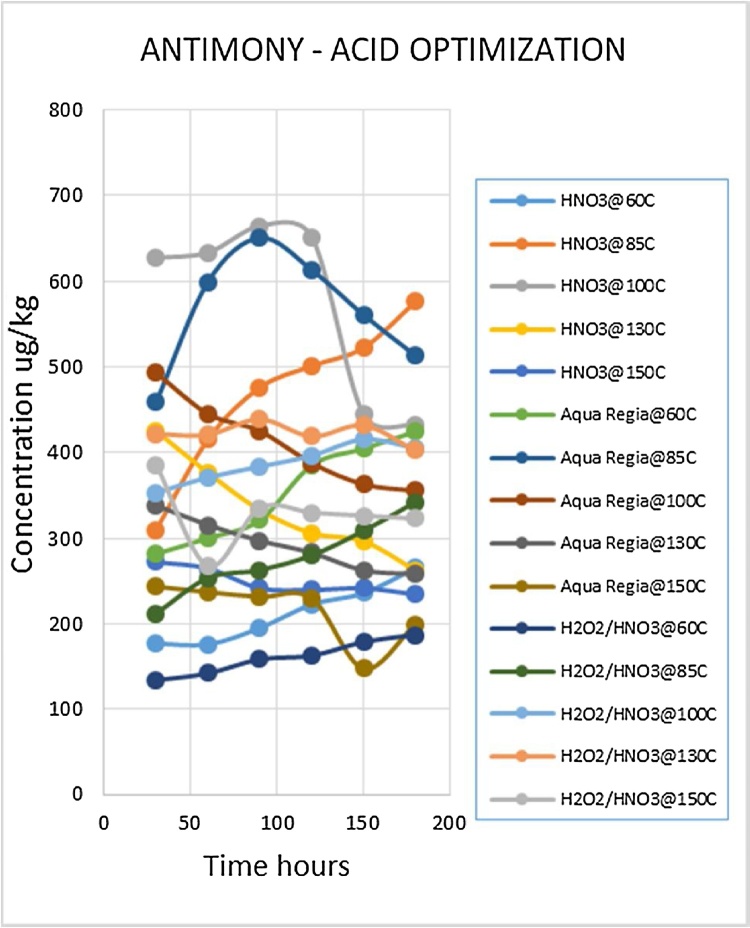
Optimization of Acid digestion for Antimony.

**Fig. 7:**
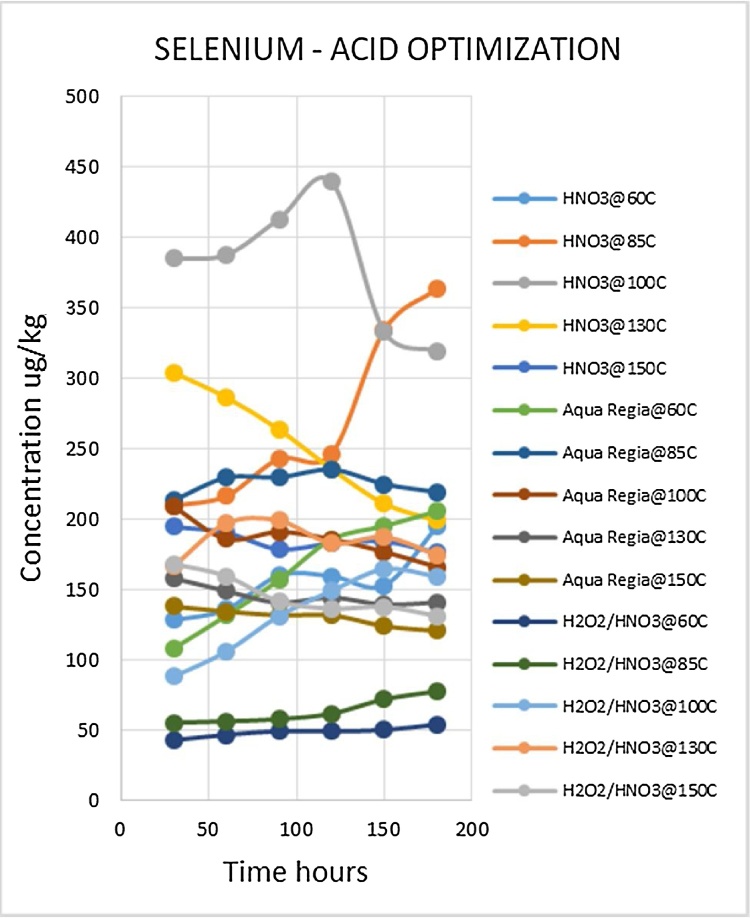
Optimization of Acid digestion for Selenium.

Heavy metal analysis of biological material requires the metals to be completely solubilized from the matrix through acid digestion. This is one of the most labor intensive and time consuming steps of the procedure. The suitable optimized conditions for each variable (digestion media, temperature and time) were selected for each metal being analyzed. Due to the large number of fish samples to be analyzed the optimized conditions that gave the best metal recoveries and could be applied to the widest range of metals was desirable. Such a procedure would reduce analysis time as well as the quantity of chemicals to be used, which lowers risks and ensures cost effectiveness. The experiment was conducted using three acid combinations while varying the digestion temperatures and times. The optimized acid for digestion of all the metals was nitric acid as shown in Fig. 2, Fig. 3, Fig. 4, Fig. 5, Fig. 6, Fig. 7 and Table 3. This was selected based on their high yielding recoveries from the shark sample as well as the DORM-4 NIST traceable standard. From the data obtained and the graphical displays of Fig. 2, Fig. 3, Fig. 4, Fig. 5, Fig. 6, Fig. 7, it is apparent that Nitric Acid at 100 °C for 120 minutes gave the most efficient extractions for the matrix studied for Selenium, Lead and Cadmium, and a very close second best for Arsenic and Antimony. Only Mercury was extracted most efficiently at the lower temperature of 85 °C for the same 120 minutes. This finding was supported by research done by [15] which suggested Nitric Acid as the most efficient digestion agent for fish muscle tissue. The digested samples were clear and transparent which indicated there was complete digestion of the sample and therefore release of the bound heavy metals from the sample matrix.

**Table 3:** A summary of the acid digestion optimized conditions selected.

Metal	Acid type	Temp/°C	Time/min	Metal recovery using Dorm-4 NIST standard	Spike Recovery
Mercury	HNO_3_	85	120	92%	94%
Arsenic		100	120	95%	97%
Antimony		100	120	95%	101%
Selenium		100	120	91%	95%
Lead		100	120	92%	97%
Cadmium		100	120	97%	98%

In keeping with the objectives of the study, the most efficient method of digestion applicable to all the metals in the study was therefore Nitric Acid at 100 °C for 120 minutes for Arsenic, Antimony, Selenium, Lead and Cadmium, and Nitric Acid at 85 °C for 120minutes for Mercury.

This finding was confirmed by the analysis of a Certified Reference Material and Spiked Recovery. These are summarized in Table 3.

The digestion procedure for nitric acid yielded good average recovery values of 91–97% for the DORM-4 reference standard, which proves that there were minimal losses of the metals due to the digestion method. The elements that were in the lower recovery range for both the DORM-4 CRM and Spikes were Selenium and Mercury which could be as a result of memory effects. This occurs when metals adhere to the surface of the vessel and does not reach the atomizer to be atomized [10]. To prevent this from occurring a longer delay time is recommended to allow all the metal to enter the atomizer. In addition, flushing the tubing with deionized water between consecutive samples could further prevent memory effects.

All the metals studied solubilized better at higher digestion temperatures with the exception of mercury which solubilized best at 85 °C. This is generally in keeping with the theory that acid digestions will yield the highest recovery at the boiling point of the acid. Also the highest yields of mercury, lead, cadmium and selenium were found at longer duration times of 2 hours (120 minutes) followed by a sudden decline in concentration. The extent of dissolution of the metals were possibly enhanced by the prolonged exposure of the sample to nitric acid. Arsenic and antimony behaved quite similarly because they both exist in the same oxidation state and yielded good recoveries in a shorter space of time compared to the other metals.

A spiked recovery analysis was performed as an additional form of quality control to calculate the recovery of the analyte spike added to the sample prior to sample preparation (Table 3). It was used to determine any matrix effects and sample preparation losses [3]. The spiked recoveries indicated that there were minimal losses through volatilization using nitric acid since the recoveries varied from a low of 94% to a high of 101%.

## Conclusion

From the recoveries of the CRM as well as the spikes, nitric acid at 100 °C for 120 minutes was the most efficient digestion protocol for the evaluation of Arsenic, Antimony, Selenium, Lead and Cadmium. For Mercury, the most efficient digestion protocol was nitric acid at 85 °C for 120 minutes. This study proves that efficient digestion of fish tissue can be achieved with minimal losses using simple controlled open acid digestions.
